# Facial nerve function and hearing after microsurgical removal of sporadic vestibular schwannomas in a population-based cohort

**DOI:** 10.1007/s00701-019-04055-4

**Published:** 2019-09-07

**Authors:** Ismail Taha, Antti Hyvärinen, Antti Ranta, Olli-Pekka Kämäräinen, Jukka Huttunen, Esa Mervaala, Heikki Löppönen, Tuomas Rauramaa, Antti Ronkainen, Juha E. Jääskeläinen, Arto Immonen, Nils Danner

**Affiliations:** 1 Neurosurgery of Neurocenter, Kuopio University Hospital and Clinical Institute, University of Eastern Finland, P.O.B 100, 70029 KYS, Kuopio, Finland; 2 Neurosurgery, Assiut University Hospital and Faculty of Medicine, Assiut University, Assiut, Egypt; 3 Otorhinolaryngology, Kuopio University Hospital and Clinical Institute, University of Eastern Finland, Kuopio, Finland; 4 Clinical Neurophysiology, Kuopio University Hospital and Clinical Institute, University of Eastern Finland, Kuopio, Finland; 5 Clinical Neuropathology, Kuopio University Hospital and Clinical Institute, University of Eastern Finland, Kuopio, Finland; 6 Neurosurgery, Tampere University Hospital, Tampere, Finland

**Keywords:** Vestibular schwannoma, Facial nerve, Hearing, Microsurgery, Retrosigmoid approach, Intraoperative monitoring

## Abstract

**Background:**

Vestibular schwannoma (VS) is a benign tumor originating from the vestibulocochlear nerve. The optimal treatment strategy is debated, since surgery may result in iatrogenic facial nerve injury. We report the results of VS surgery in a population-based unselected cohort in a center with access to Cyber Knife (CK) radiosurgery.

**Methods:**

We reviewed 117 consecutive operations and found 95 patients who had their primary operation due to vestibular schwannoma between 2001 and 2017. Facial nerve function was evaluated with the House-Brackmann (HB) scale and hearing with the EU classification.

**Results:**

The population consisted of 37 males and 58 females with a median age of 54 years (range 19–79). One year after surgery 67% of patients had a good outcome (HB 1–2). The rate of good outcome was 90% if no facial nerve damage was observed during intraoperative monitoring, the size of the tumor was under 30 mm and no hydrocephalus was present. During the study period, the treatment strategy changed from total to near-total resection after the introduction of CK radiosurgery, which could be used as a second-line treatment in case of residual tumor regrowth. This resulted in an improvement of outcomes (0% HB 5–6) despite the larger tumor sizes (25 ± 14 mm vs. 31 ± 9 mm, *p* < 0.05). Hearing preservation rates did not increase.

**Conclusions:**

Near-total resection and subsequent CK radiosurgery in case of residual tumor regrowth during follow-up seems to provide a good outcome of facial nerve function even in large VSs.

## Introduction

Vestibular schwannoma (VS), previously also called acoustic neuroma, is a benign, slow-growing tumor originating from the Schwann cells of the vestibular branch of the vestibulocochlear nerve [17]. The annual incidence of VS is 1–2:100,000 making it the third most common benign intracranial tumor. In its location, the cerebellopontine angle (CPA), VS is the most common type of tumor [25, 30, 37, 50]. The typical symptoms of VSs are caused by compression on the adjacent cranial nerves and may present as hearing loss, tinnitus, dizziness, facial numbness or weakness. Large VSs may even cause hydrocephalus or brainstem compression [14, 31]**.** Sporadic VSs are almost exclusively unilateral, whereas bilateral VSs are typically associated with neurofibromatosis type 2 (NF2) [3].

Since VSs are benign of nature, their treatment options are dependent on the symptoms caused by the tumor. Small VSs with mild symptoms may be followed-up by repeated MR-imaging, whereas larger tumors with pronounced symptoms or tumors with a rapid growth rate may warrant aggressive treatment. Treatment indications and modalities for VSs vary between different centers. The aim of VS surgery is maximum safe resection without causing additional neurological defects in the function of adjacent cranial nerves. In this perspective, the preservation of facial nerve function is crucial due to its location in the immediate proximity of the tumor. However, surgical treatment of VSs results in permanent facial weakness in 10–40% of patients [15, 54, 63]. In order to minimize iatrogenic nerve injuries, intraoperative neurophysiological monitoring and direct nerve stimulation have become routine practice to aid the recognition and preservation of the cranial nerves during surgery [1, 43, 58, 60]. Since small tumors have a better prognosis for facial nerve preservation after surgery some centers opt for an aggressive treatment scheme, whereas a more conservative approach with regular follow-ups allows the treatment to be targeted to tumors with growth tendency avoiding unnecessary surgeries and operative complications. Furthermore, the extent of resection is a matter of debate, since gross total resection (GTR) may impose a greater risk on the function of the facial nerve, whereas near-total (NTR) or subtotal resection (STR) may lead to tumor recurrence requiring reoperation or radiation therapy [16, 44, 45, 50].

The aim of the current study is to evaluate facial nerve function in a population-based series of consecutive patients operated for VS with intraoperative neurophysiological monitoring between the years 2001 and 2017. During the study period, the surgical strategy shifted from total to near-total resection with the aim of avoiding iatrogenic facial nerve paresis. The introduction of stereotactic Cyber Knife (CK) radiosurgery (Accuracy Inc., Sunnyvale, CA) led to a further paradigm shift in the treatment protocol by providing a second-line treatment option in the case of residual tumor growth. The purpose of the present study is to characterize factors, which influence the functional outcome of the facial nerve after VS surgery via the retrosigmoid approach in a center with routine intraoperative monitoring and access to CK radiosurgery.

## Patients and methods

### Literature review

PubMed was searched for original publications in the English language from the year 2000 onwards with the following search words: (vestibular or acoustic or acustic) and (schwannoma* or neuroma* or neurinoma*) and ((facial or seventh) and nerve)) and (monitoring or stimulation or mapping). Case reports, abstracts and articles with insufficient outcome data were further excluded. The results of all 29 relevant original publications reporting long-term results of over 100 patients are presented in Table 1 [2, 5–10, 18, 20–23, 26–29, 33, 34, 36, 40, 44, 46, 48, 49, 52, 55, 56, 61, 62].

**Table 1:** Previous original publications from the year 2000 onwards reporting long-term results of studies with over 100 patients

Reference (1st author country, year)	Number of patients	Age (years)	Gender	Tumor size	Approach	Extent of resection	Preserved anatomical facial nerve integrity	Functional outcome (HB grading)
Chang S Canada 2019 [8]	434	49.1 (13–81) (mean (range))	m 213 f 221	26 ± 1 mm (mean ± SD)	RSA 85% TLA 7.3% MFA 3.9%	GTR 83.0% NTR 9.0% STR 8.1%	n.a.	G 1–2 96% G 3–6 4%
Huang X China 2018 [22]	103	47.5 (22–71) (mean (range))	m 48 f 55	32 mm (mean)	n.a.	n.a.	Only intact included	G 1–2 76% G3–6 24%
Hong WM^a^ China 2017 [18]	105 (IOM used in 83.1%)	48.7 (14–73) (mean (range))	m 41 f 64	90.5% ≥ 30 mm	RSA	GTR 80.9% STR 14.3% PR 4.8%	95.3%	G 1–2 74.4%
Boublata L Algeria 2017 [7]	151	48.2 (17–78) (mean (range))	m 43 f 98	31–60 mm	RSA	GTR/NTR 82.6% STR 13.9% PR 3.3%	98.7%	G 1–2 82% G 3–4 12% G 5–6 6%
Huang X China 2017 [21]	657 (IOM used in 81.2%)	46.8 (12–80) (mean (range))	m 368 f 289	> 40 mm	RSA	GTR 84.6% NTR 15.1% PR 0.3%	89.6%	G 1–2 55.8% G 3 19.8% G 4–6 24.4%
Huang X China 2017 [20]	1167 (IOM used in 82%)	47.5 (12–80) (mean (range))	m 535 f 632	> 30 mm	RSA	GTR 86.2% STR 13.6% PR 0.2%	92.8%	G 1–2 87.9% G 3–4 11.6% G 5–6 0.3%
Torres R France 2017 [53]	229	49 (15–84) (mean (range))	m 92 f 137	61% < 15 mm 7% > 30 mm	TLA 78% RSA 21% MFA 1%	n.a.	n.a.	G 1–2 84% G 3–4 15% G 5–6 1%
Bhimrao S Canada 2016 [6]	367	49 (13–81) (mean (range))	m 178 f 189	26 ± 10 mm (mean ± SD)	RSA 87% TLA 10% MFA 3%	STR 9%	n.a.	G 1–2 95.2% G 3–4 4.2% G 5–6 0.6%
Kunert P Poland 2016 [25]	212	≤ 50, *n* = 121 > 50, *n* = 91	m 83 f 129	30 mm (mean) 56% ≤ 30 mm 44% > 30 mm	RSA 99% TLA 1%	GTR 99% NTR 1%	84–94%	G 1–3 77% G 4–6 23%
Nejo T^b^ Japan 2016 [35]	556 D: 21 ND 535	46 (11–78) (median (range))	m 246 f 310	D: 28 mm (10–45) ND: 24 mm (0–64) (mean (range))	RSA	GTR or NTR D: 38.1% ND: 85.4%	D:100% ND: 99.4%	G1–2 D: 95.2% ND: 97%
Zhang J China 2015 [58]	221	46.1 (29–73) (mean (range))	m 105 f 116	82.8% ≥ 30 mm 17.2% < 30 mm	RSA	NTR 90% STR 10%	n.a.	G1–3 82.8% G4 7.2% G5 10%
Liu SW^c^ China 2015 [26]	106	48 (19–76) (mean (range))	m 40 f 66	≥ 30 mm	RSA	GTR 82.1% STR 14.2% PR 3.7%	98.1%	G1–2 79.3% G3 20.8% G4 0.9%
Spektor S Israel 2015 [46]	130	44.3 (14–83) (mean ((range))	m 54 f 76	30 ± 11 (10–60) (mean ± SD (range))	n.a.	GTR 76.1% STR 20.0% PR 3.9%	n.a.	G1–2 83.1% G3–4 13.1% G5–6 4.7%
Porter R USA 2013 [38]	153 (63 single surgery, 75 staged surgery)	55.8 (13–83) in single 46.4 (17–80) in staged (mean (range))	m 82 f 71	≥ 30 mm	TLA in single, followed by RSA in staged	In single surgery: GTR 46% NTR 30% STR 24%	n.a.	G1–2 75% in single 81% in staged
Schmitt W 2013 USA [44]	267	48 (15–86) (mean (range))	m 120 f 147	24 mm (8–60 mm) (mean (range))	RSA 58.5% TLA 34% MFA 7.5%	GTR 75% NTR 12% STR 13%	Only intact included	G1–2 84%
Marin P 2011 Canada [28]	106	50.4 (20–78) (mean (range))	m 63 f 43	17.5 mm (average)	TLA 61% RSA 38% both 1%	n.a.	Only intact included	G1–2 95%
Amano M 2011 Japan [2]	216	45.1 (14–76) (mean (range))	m 104 f 112	25 mm (0–55 mm) (mean (range))	RSA	resection rate 98.2% (85–100%) (mean (range))	100%	G1–2 98.6%
Morton R^d^ 2011 USA [33]	104	39.6 (13–71) (mean (range))	m 53 f 51	DFP 20.8 ± 8.6 mm IFP 32.2 ± 16.0 mm no FP 21.2 ± 16.0 mm (Mean ± SD)	RSA 47.1% TLA 41.3% MFA 2.9% RSA+TLA 8.7%	n.a.	n.a.	G1–2 97.1%
Sughrue M 2010 USA [49]	477	49–51 (range of means)	m 223 f 245	20–30 mm (range of means)	TLA 50% MFA 22.8% RSA 22.8%	GTR 69.2% NTR 14.0% STR 16.8%	n.a.	G1–2 57.4% G3–6 42.6%
Chen L 2010 China [10]	145	42.3 (22–71) (mean (range))	m 77 f 68	82% > 30 mm 18% ≤ 30 mm	RSA	GTR 96.6%	91%	G1–2 79.3%
Bernat 2010 France [5]	120	50 (23–83) (mean (range))	m 53 f 67	60% ≤ 15 mm 12.5% > 30 mm	TLA 78% MFA 3% RSA 13% Transotic 6%	n.a.	n.a.	G1–2 69% G 3–4 18% G 5–6 13%
Chen L 2009 China [9]	103	45.1 (19–76) (mean (range))	m 45 f 58	38 mm (15 – 67 mm) (mean (range))	RSA	GTR 98.1% STR 1.9%	98.1%	G1–2 83.5% G3–4 17.5%
Shamji M 2007 Canada [45]	128	n.a.	n.a.	23 mm (5–70 mm) (average (range))	TLA	n.a.	n.a.	G1–2 87%
Samii M^e^ 2006 Germany [42]	200	46.8 (18–73) (mean (range))	n.a.	T1 11%, T2 9%, T3a 14%,T3b 20%, T4a 36%, T4b 10%	RSA	GTR 98% STR 2%	98.5%	G1–2 81%
Meyer T 2006 USA [32]	162	49 (19–70) (mean (range))	m 83 f 79	2–25 mm (range)	MFA	GTR 100%	100%	G1 86.4% G2 10.5% G3 3.1%
Zhang X 2005 China [59]	105	46.8 (21–75) (mean (range))	m 41 f 64	> 40 mm	RSA	GTR 86.7% STR 13.3%	79.1%	G1–2 56.7% G3–4 21.8% G5–6 21.9%
Isaacson B 2003 USA [23]	229	51 (15–79) (mean (range))	m 114 f 115	19 mm (4 – 65 mm) mean (range)	TLA 59% RSA 29% MFA 12%	n.a.	97%	G1–2 87%
Magnan J 2002 France [27]	119	n.a.	n.a.	< 25 mm	RSA	n.a.	100%	G1–2 96%
Tonn J-C 2000 Germany [52]	508 (IOM 396, 80%)	51.3 (14–80) (mean (range))	m 263 f 245	8–40 mm (range)	RSA	n.a.	n.a.	G1–2 With IOM 88.7% Without IOM 69.5%

*RSA* retrosigmoid approach, *TLA* translabyrinthine approach, *MFA* medial fossa approach, *GTR* gross-total resection, *NTR* near-total resection, *STR* sub-total resection, *PR* partial resection, *IOM* intraoperative monitoring

^a^26% loss of 1-year follow-up

^b^Results are divided according to the location of the facial nerve with respect to the tumor as dorsal (D) or non-dorsal (ND)

^c^43% loss of late follow-up

^d^IFP (immediate facial palsy); DFP (delayed facial palsy)

^e^Hannover classification

### Patients

A population-based cohort was retrospectively collected from the catchment area of Kuopio University Hospital comprising the population of Central and Eastern Finland with over 830,000 people (Statistics Finland). All patients were operated at the Neurocenter of Kuopio University Hospital. The operation journals and pathology databases were screened to find all operatively treated VSs between the years 2001 and 2017. The files of all patients were thoroughly reviewed and the MRIs re-evaluated. Tumor sizes were measured and classified according to the Koos grading [24]. Pre- and postoperative audiograms were re-evaluated and hearing was graded according to the EU classification [57]. Facial nerve function was evaluated according to the House-Brackmann scale [19].

### Treatment protocol

All patients in the study were operated via the retrosigmoid approach. Neurophysiological monitoring was used to guide safe tumor resection. Continuous EMG-monitoring of V and VII cranial nerves was performed and direct nerve stimulation was routinely applied in all elective operations. Depending on the size of the tumor and the clinical situation, IX–XII cranial nerves were also monitored. The monitoring was performed by an experienced clinical neurophysiologist present at the operation. The monitoring system and setup varied during the study period and has been adjusted individually when needed. The surgical strategy was changed in case spontaneous EMG activity indicative of facial nerve damage was observed or if the stimulation threshold for eliciting motor evoked potentials increased [43]. In case the internal acoustic meatus was drilled in order to remove the intrameatal part of the tumor, an experienced surgeon in otorhinolaryngology was attending the operation in addition to the neurosurgeon.

From 2013 onwards, stereotactic CK radiosurgery was adapted as a part of the treatment protocol of VSs at Kuopio University Hospital. Between the years 2001 and 2012 (pre-CK era) the aim of surgery was gross-total resection of the tumor with drilling of the internal acoustic channel in order to completely remove also the intrameatal part of the tumor (pre-CK era). In the latter part of the study period, during the years 2013–2017 (post-CK era), the emphasis was shifted towards preserving the function of the facial nerve leading to a treatment strategy with near-total resection of the tumor [38, 47]. A thin layer of tumor tissue was intentionally left on the adherent parts of the facial nerve and the internal acoustic meatus was not drilled. In case of residual tumor regrowth during subsequent follow-up, CK radiosurgery was used as second-line treatment option.

### Follow-up

Facial nerve function was followed up 12 months after the operation. Tumor residual and regrowth was evaluated by repetitive MRI scans. Data on mortality, reoperation, or adjuvant treatment with radiosurgery was collected until the end of the study period (follow-up time up to 16 years).

### Statistical methods

For statistical analysis, nonparametric tests (Mann-Whitney, chi^2^, and Kruskal-Wallis) were used. For statistical significance, the *p* level was set at 0.05. A classification tree analysis was performed to evaluate the effect of patient-, tumor-, and treatment-related factors on the final outcome.

### Ethical aspects

The study protocol was approved by the ethics committee of Kuopio University Hospital.

## Results

**Fig. 1:**
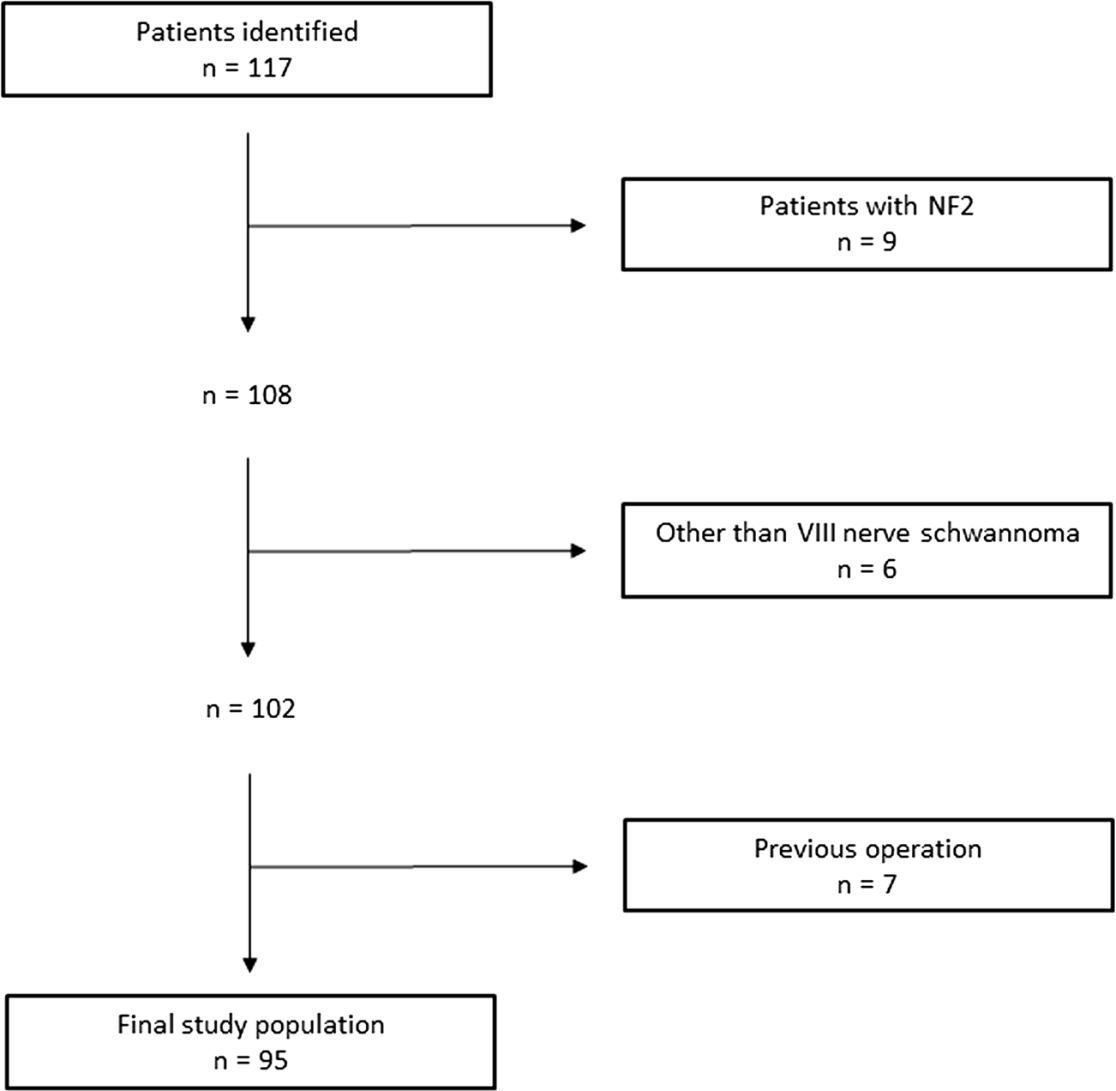
Flow chart of the patient recruitment for the study

A total number of 117 patients were operated during the study period. A flow chart of the patient recruitment is presented in Fig. 1. Nine patients with neurofibromatosis type 2, six patients with schwannomas of other than VIII cranial nerve were excluded from the analyses. Seven patients had had a previous operation before the beginning of the study period and were therefore excluded from the study. This resulted in a final study population of 95 patients with 37 males and 58 females with a median age of 54 years (range 19–79 years).

There was no perioperative mortality prior to discharge. Two patients died within 3 months following the operation and further two within 1 year. Three patients were lost before the end of the 1-year follow-up period.

**Fig. 2:**
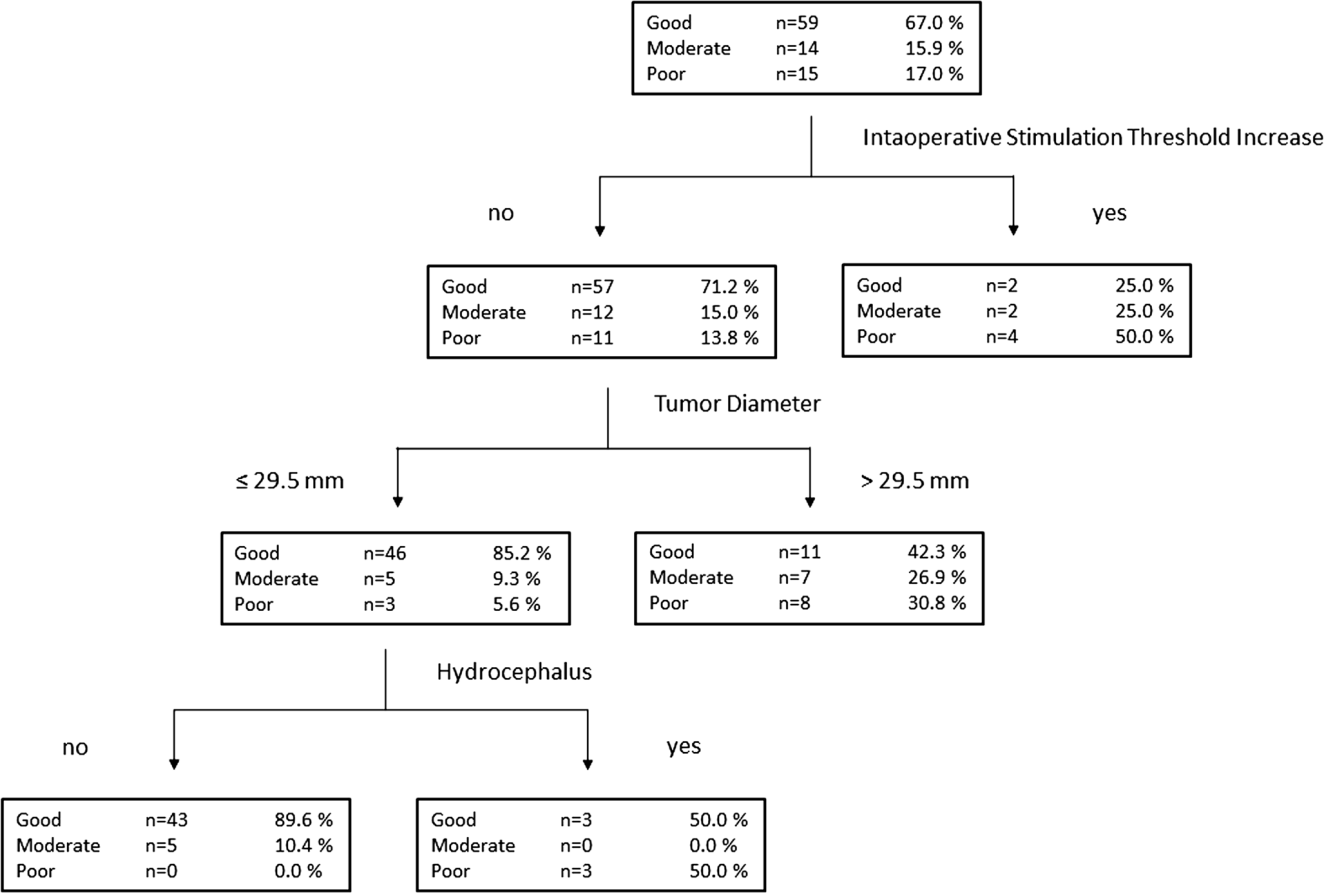
Classification tree analysis of outcome at 1 year after vestibular schwannoma surgery

**Fig. 3:**
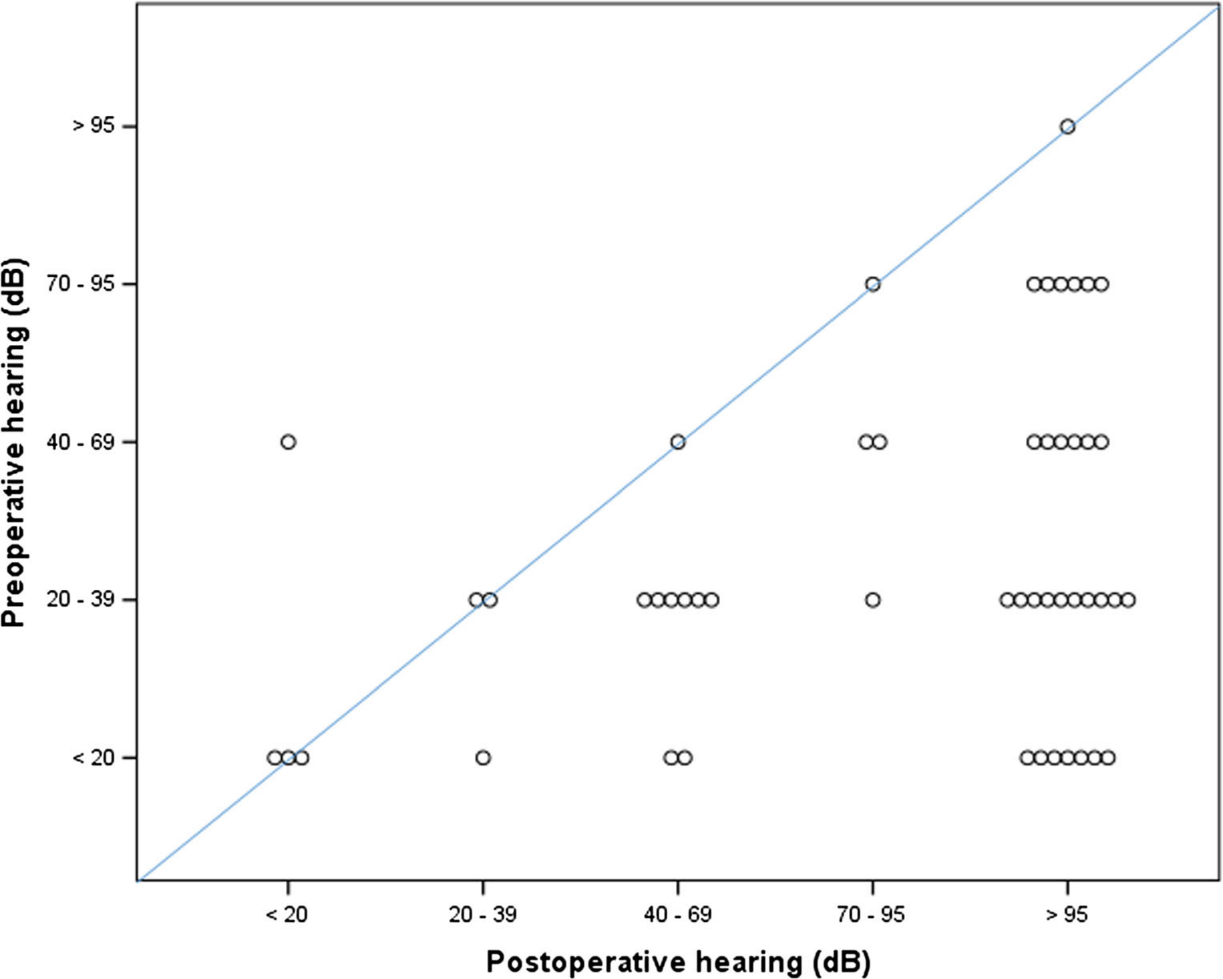
Hearing in the ipsilateral ear of individual patients in decibels (dB) corresponding to the hearing classes (1–5) before and after vestibular schwannoma surgery

The outcomes of the patients are presented in Table 2. In the total population, 67% of the patients had a good outcome (HB 1–2), 16% a moderate outcome (HB 3–4) and 17% a poor outcome (HB 5–6). Tumor sizes, diameters, and volumes differed significantly between the outcome groups (*p* < 0.05). In all patients with a poor outcome, the tumor was in contact with the brainstem or even compressing it. However, mean the Koos grades did not differ significantly between the outcome groups. The symptomatology of the patients showed also significant differences between the groups. Patients who exhibited a worse clinical outcome had more commonly clinical signs of brainstem and cerebellar compression as well as preoperative facial nerve dysfunction (*p* < 0.05). The extent of resection and complication rate did not differ between the outcome groups. However, in the poor outcome group, intraoperative neurophysiological monitoring indicated facial nerve damage in 40% of patients (*p* < 0.05). A classification tree analysis of outcomes is presented in Fig. 2. Preoperative audiograms were available for 63 patients and postoperative audiograms for 65 patients. Normal preoperative hearing (class I, < 20 dB) was found in 23.8% of patients and 7.9% of patients were deaf on the affected side. Postoperatively, the hearing class remained unchanged in 16.0% of patients and improved in one patient. A decrease of two or more hearing classes was observed in 52.0% of patients and 66.2% were deaf (class V, > 95 dB) on the operated side. The distribution of pre- and postoperative hearing is illustrated in Fig. 3 as decibels corresponding to the hearing classes.

**Table 2:** Differences in clinical and operative parameters according to the outcome of facial nerve function after vestibular schwannoma surgery

	Good outcome (HB 1–2) *n* = 59 (67%)	Moderate outcome (HB 3–4) *n* = 14 (16%)	Poor outcome (HB 5–6) *n* = 15 (17%)	Difference between groups
Age (mean ± SD)	51 ± 13 years	55 ± 16 years	55 ± 15 years	
Gender (male/female)	20/39	7/7	6/9	
Size^1^				p < 0.05
Intrameatal	2 (4.1%)	1 (7.1%)	1 (7.7%)	
Small	6 (12.2%)	1 (7.1%)	–	
Medium	31 (63.3%)	4 (28.6%)	5 (38.5%)	
Large	10 (20.4%)	8 (57.1%)	7 (53.8%)	
Largest diameter^2^ (mean ± SD, range)	24 ± 12 mm 0–53 mm	33 ± 10 mm 0–51 mm	32 ± 15 mm 0–50 mm	p < 0.05
Volume^2^ (mean ± SD, range)	9000 ± 11,000 mm^3^ 26–49,000 mm^3^	16,000 ± 11,000 mm^3^ 3200–34,000 mm^3^	17,000 ± 21,000 mm^3^ 2700–47,000 mm^3^	p < 0.05
Koos grade (mean ± SD, range)	3.4 ± 0.9 1–4	3.6 ± 0.6 2–4	3.8 ± 0.4 3–4	
Clinical presentation				
Hydrocephalus	7 (11.9%)	4 (28.6%)	5 (33.3%)	
Headache	9 (15.3%)	4 (28.6%)	5 (33.3%)	
Vertigo	35 (59.3%)	6 (42.9%)	6 (40.0%)	
Imbalance/ataxia	12 (20.3%)	5 (35.7%)	9 (60.0%)	p < 0.01
Hearing	51 (86.4%)	11 (78.6%)	14 (93.3%)	
Tinnitus	19 (32.2%)	3 (21.4%)	6 (40.0%)	
Facial	1 (1.7%)	2 (14.3%)	3 (20.0%)	p < 0.05
Trigeminal	15 (25.4%)	3 (21.4%)	2 (13.3%)	
Brainstem/cerebellar	5 (8.5%)	1 (7.1%)	5 (33.3%)	p < 0.05
Hearing class^3^ (mean, range)				
Preoperative	2.4 (1–5)	2.6 (1–3)	3.3 (2–5)	
Postoperative	4.1 (1–5)	4.2 (1–5)	5.0 (5)	
Change	1.8	1.6	2.3	
Extent of resection				
Gross total	17 (29.3%)	4 (28.6%)	5 (33.3%)	
Near total	35 (60.3%)	10 (71.4%)	7 (46.7%)	
Subtotal	6 (10.3%)	–	2 (13.3%)	
Partial	–	–	1 (6.7%)	
Drilling^4^	28 (48.3%)	7 (50.0%)	7 (46.7%)	
Threshold increase^5^	2 (2.3%)	2 (16.7%)	4 (66.7%)	p < 0.001
Loss of response^5^	–	2 (15.4%)	3 (37.5%)	p < 0.01
Complication^6^	4 (6.8%)	2 (14.3%)	4 (26.7%)	
Regrowth^7^	12 (20.3%)	3 (23.1%)	6 (40.0%)	

Differences between groups were tested using the Kruskall-Wallis test and the chi square test. Significant differences have been indicated. Valid percentages of available data are reported for each parameter

^1^Size is determined according to largest extrameatal diameter (small ≤ 15 mm, 15 mm < medium ≤30 mm, large >30 mm)

^2^Diameter and volume are calculated for the extrameatal part of the tumor

^3^Koos grading of vestibular schwannoma: 1, intrameatal; 2, extending to the cerebellopontine angle; 3, in contact with the brainstem; 4, compressing the brainstem

^4^Hearing is determined according to the WHO classification

^5^Drilling of internal acoustic meatus

^6^Intraoperative increase in stimulation threshold or loss of response in facial nerve monitoring

^7^Postoperative complication requiring re-operation

^8^Regrowth during subsequent follow-up leading to intervention

Seventy-three patients were operated before the introduction of CK and 22 during the CK era (Table 3). Patient demographics did not differ between the groups. In the CK era, the extent of resection was targeted to the extrameatal part of the tumor in over 91% of cases as opposed to the pre CK era when the also intrameatal part of the tumor was removed in 58% of cases (*p* < 0.05). In the CK era, the diameters and volumes of the operated tumors were larger (*p* < 0.05) and all had a Koos grade 3 or 4. Despite the larger tumor sizes, the facial nerve outcomes were better (*p* < 0.05) as compared to the pre CK era and there were no poor outcomes.

**Table 3:** Clinical parameters and outcomes of vestibular schwannoma surgery before and after treatment paradigm change with the introduction of Cyber Knife (CK) radiosurgery

	Before CK (*n* = 73) 2001–2012	After CK (*n* = 22) 2013–2017	
Age (mean ± sd)	53 ± 14 years	52 ± 16 years	
Gender (male/female)	30/43	7/15	
Diameter (mean ± sd)	25 ± 14 mm	31 ± 9 mm	p < 0.05
Volume (mean ± sd)	10,400 ± 12,100 mm^3^	14,100 ± 11,400 mm^3^	p < 0.05
Size^1^			
Intrameatal	6.6% (*n* = 4)	–	
Small	11.5% (*n* = 7)	–	
Medium	50.8% (*n* = 31)	54.5% (*n* = 12)	
Large	31.1% (*n* = 19)	45.5% (*n* = 10)	
Koos grade (mean ± sd (range))^2^	3.32 ± 0.81 (1–4)	3.95 ± 0.21 (3–4)	
Extent of resection			p < 0.01
Gross total	36.1% (*n* = 26)	9.1% (*n* = 2)	
Near total	47.2% (*n* = 34)	90.9% (*n* = 20)	
Subtotal	13.9% (*n* = 10)	–	
Partial	2.8% (*n* = 2)	–	
Drilling^3^	58.3% (*n* = 42)	9.1% (*n* = 2)	p < 0.001
Complication^4^	15.1% (*n* = 11)	4.5% (*n* = 1)	
Regrowth^5^	24.7% (*n* = 18)	19.8% (*n* = 4)	
Facial nerve outcome^6^			p < 0.05
Good	65.2% (*n* = 43)	68.4% (*n* = 13)	
Moderate	12.1% (*n* = 8)	31.6% (*n* = 6)	
Poor	22.8% (*n* = 15)	–	
Hearing class (mean ± SD) ^7^			
Preoperative	2.5 ± 1.2	2.6 ± 1.5	
Postoperative	4.2 ± 1.3	4.3 ± 0.9	
Change	1.7 ± 1.4	2.0 ± 1.5	

Significant differences have been indicated according to the Mann-Whintey *U* and chi square test

^1^Size is determined according to largest diameter (small ≤ 15 mm < medium ≤ 30 mm < large)

^2^Koos grading of vestibular schwannoma: 1, intarmeatal; 2, extending to the cerebellopontine angle; 3, in contact with the brainstem; 4, compressing the brainstem

^3^Drilling in internal acoustic meatus

^4^Complication requiring re-operation

^5^Regrowth leading to reoperation or radiosurgery

^6^Facial nerve outcome at 12 months by House-Brackman grade (1–2 good, 3–4 moderate, 5–6 poor)

^7^Hearing is determined according to the WHO classification

## Discussion

This study presents the outcomes of patients operated due to vestibular schwannoma in a population-based non-selected cohort. All in all, 67% of patients achieved a good facial nerve outcome. According to the classification tree analysis, a good outcome rate of 90% was achieved if no stimulation threshold increase was observed during intraoperative monitoring, the size of the tumor was under 30 mm and no hydrocephalus was present (Fig. 2.). In a regression analysis, none of the parameters was found to be independently associated with either a good or a bad outcome. However, patients with a less favorable outcome had significantly larger tumors in terms of diameter and volume (Table 2.). In accordance, preoperative symptoms of brain stem compression due to larger tumors were more commonly associated with poor facial nerve outcomes. Unsurprisingly, preoperative facial nerve dysfunction was also more common in patients with a poor postoperative result. The results of the current study are in line with previous literature in terms of the postoperative facial nerve preservation rate, which decreases considerably with tumor size (Table 1).

Neuromonitoring with facial nerve stimulation has become an essential part of VS surgery during the past decades [1, 58]. The use of monitoring has been shown to improve the outcome of surgery and, when indicating nerve damage, to have also a predictive value in terms of operative outcome [2, 4, 29, 52]. In the current study, this predictive value was evident, since in the good outcome group, only two patients exhibited an intraoperative stimulation threshold increase, whereas nearly 70% of patients in the poor outcome group showed signs of facial nerve damage during surgery (Table 2 and Fig. 2). In addition to direct facial nerve stimulation, the pattern of spontaneous EMG activity during the operation has also been applied to guide tumor resection and shown to obtain predictive value. However, the interpretation of spontaneous EMG activity is more complex, since only specific wave forms and trains have been reported to associate to FN damage [35, 41, 43]. Spontaneous EMG activity was not evaluated in the current study, since the actual recording data was not available in retrospect.

Hearing impairment is the most common initial symptom of VS and the natural course has been shown to lead to non-seviceable hearing in 50% of patients over a 5-year period in patients with good initial hearing [39, 42, 53]. Accordingly, hearing is impaired in most patients already before surgery. However, in patients with good initial hearing and small tumor size, up to over 60% have been reported to have good or serviceable postoperative hearing [32], whereas in larger tumors the percentage is far lower especially if hearing is affected already preoperatively [59]. In the current study, hearing was impaired in most patients already preoperatively and only 23.8% had normal hearing on the affected side. Postoperatively, 18% of patients retained or improved their hearing class and 66.2% were deaf. Hearing outcome did not correlate with facial nerve outcome (Table 2 and Fig. 3). Thus, in large VSs, operative treatment cannot be justified with the aim of hearing preservation.

Since total resection of especially large VSs carries a considerable risk of facial nerve injury and hearing defect, a less radical treatment paradigm has been introduced during the recent years. After planned sub-total surgical resection combined with adjuvant radiosurgery, better outcomes have been reported in terms of hearing and facial nerve preservation as well as tumor control [11–13, 51]. During the current study period, the introduction of CK radiosurgery changed the treatment protocol of VSs also at our institution. The operative paradigm shifted to near-total resection and subsequent CK radiosurgery in the case of regrowth during follow-up. This resulted in significantly improved postoperative facial nerve outcomes, since no poor outcomes were seen after the paradigm shift despite the larger tumor sizes (Table 3). In addition, the immediate postoperative complication rate was lower, although not statistically significant. However, in the current study population the change in the treatment paradigm did not improve hearing outcomes. The tumor regrowth percentage was rather similar in the pre- and post-CK era. This may suggest a higher regrowth percentage after near-total resection, since the follow-up period was shorter for the patients treated during the latter period. However, the availability of second-line CK-radiosurgery may have led to treatment of smaller-sized tumor regrowth as compared to the pre-CK era. Furthermore, in addition to the difference in the length of the follow-up, the number of patients operated during the CK era was less than one third that in the pre CK era, and therefore no definite conclusions can yet be drawn on the regrowth rate of the residual tumor.

## Conclusions

In this study we present the facial nerve and hearing outcomes after VS surgery in an unselected population-based cohort. An operative treatment paradigm with near-total resection of the tumor and subsequent CK radiosurgery in case of residual tumor regrowth during follow-up seems to result in a better functional outcome of the facial nerve.
